# Supplementary material for: Loss of PRC1 induces higher-order opening of Hox loci independently of transcription during Drosophila embryogenesis
Source: Nat Commun. 2018 Sep 25;9:3898. doi: 10.1038/s41467-018-05945-4 (PMC6156336; doi:10.1038/s41467-018-05945-4)
Supplement: Supplementary file 1 — Supplementary Information [file 41467_2018_5945_MOESM1_ESM.pdf]

Supplementary Information

Loss of PRC1 induces higher-order opening of Hox loci independently of transcription during *Drosophila* embryogenesis

Thierry Cheutin and Giacomo Cavalli

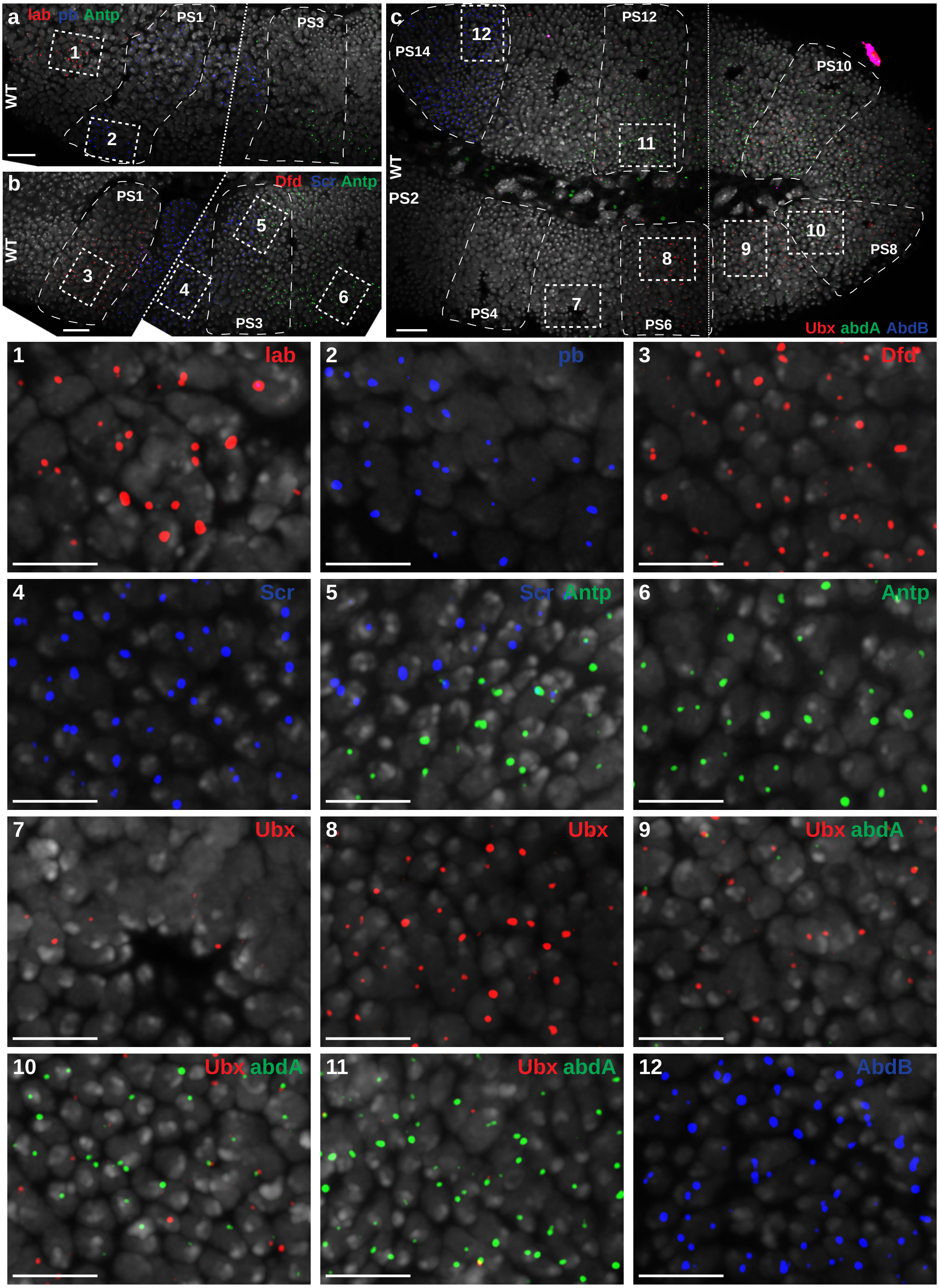

Supplementary Figure 1: Hox genes expression in WT embryos. a-c: RNA FISH pictures of wild-type (WT) *Drosophila* embryos 3:50-7:20 after fertilization illustrating the pattern of Hox gene transcription. These images are maximum intensity projections of confocal images and dash lines indicate where two images were merged. Scale bars, 20 µm. PS, parasegment. 1-12: insets focusing a few cells showing nuclear RNA-FISH spots along the anteroposterior axis. Scale bars, 10 µm.

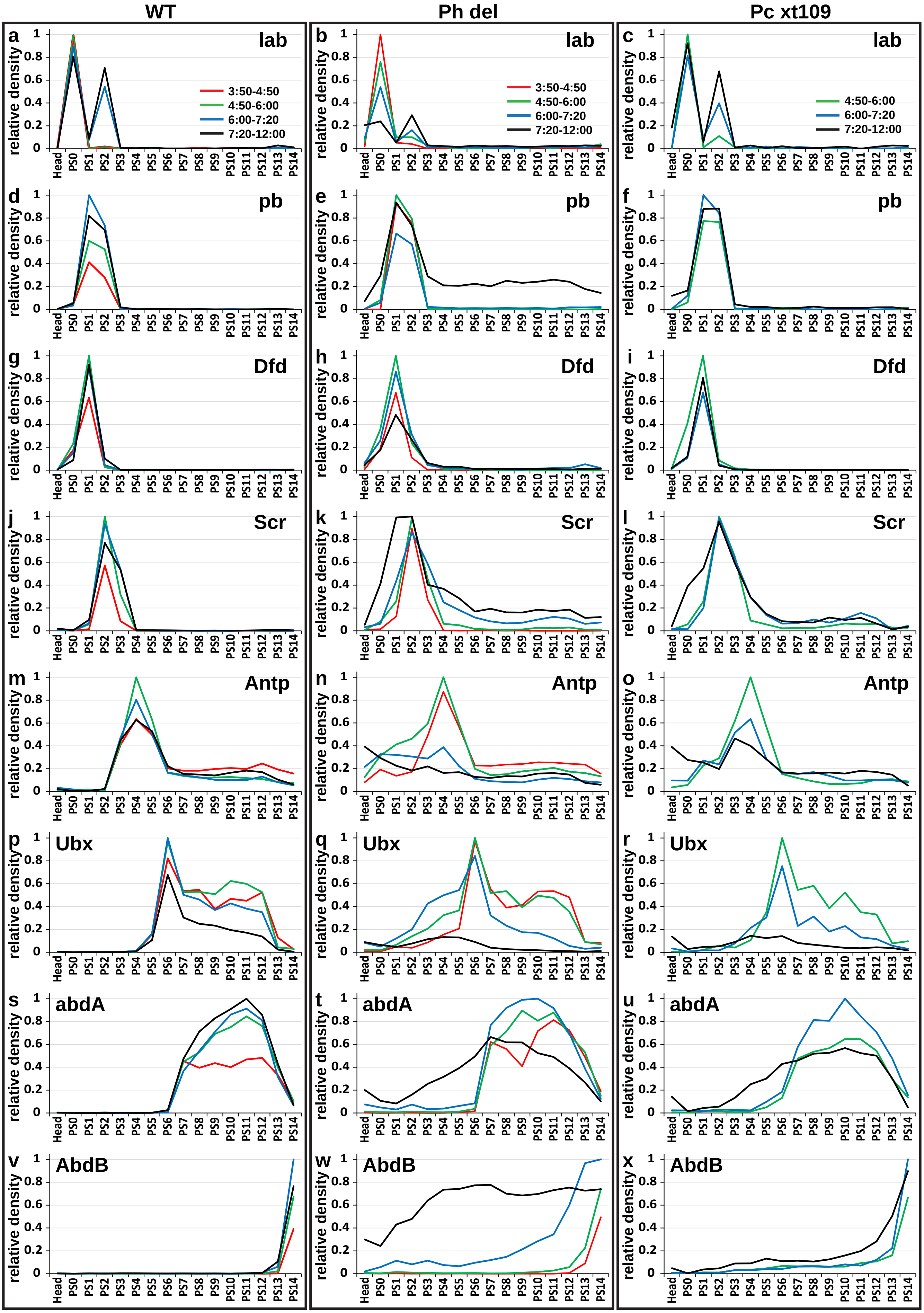

Supplementary Figure 2: Hox genes expression in WT, Phdel and PcXT109 embryos. Relative density

of RNA FISH spots measured for each Hox gene (*lab*: a-c; *pb*: d-f; *Dfd*: g-i; *Scr*: j-l; *Antp*: m-o; *Ubx*: p-r; *abdA*: s-u; *AbdB*: v-x) along the anteroposterior axis of WT (a, d, g, j, m, p, s, v), ph^{del} (b, e, h, k, n, q, t, w) and Pc^{XT109} (c, f, i, l, o, r, u, x) embryos. Time course quantification shows that Hox gene expression changes during embryogenesis (3:50-4:50: red; 4:50-6:00: green; 6:00-7:20: blue and 7:20-12:00: black) in both ph^{del} and Pc^{XT109} embryos. The first ectopic Hox gene expression observed in ph^{del} embryos occurs as soon as 3:50-4:50, whereas it appears only at 4:50-6:00 in Pc^{XT109} embryos. The proportion of cells showing ectopic Hox gene expression increases globally during the later embryogenesis of both ph^{del} and Pc^{XT109} embryos. Ph deletion generally has a stronger effect on Hox gene deregulation than Pc deletion, except for ectopic expression of *AbdB* in PS3-12 and *abdA* in PS5-6 at 4:50-6:00. Although early effects of Ph and Pc always showed Hox gene derepression, we observed *Ubx*, *abdA* and *Antp* silencing during late embryogenesis in both mutants. This late Hox genes silencing most likely reflects an indirect effect because posterior Hox genes can silenced the anterior ones, a phenomenon defined as "posterior dominance".

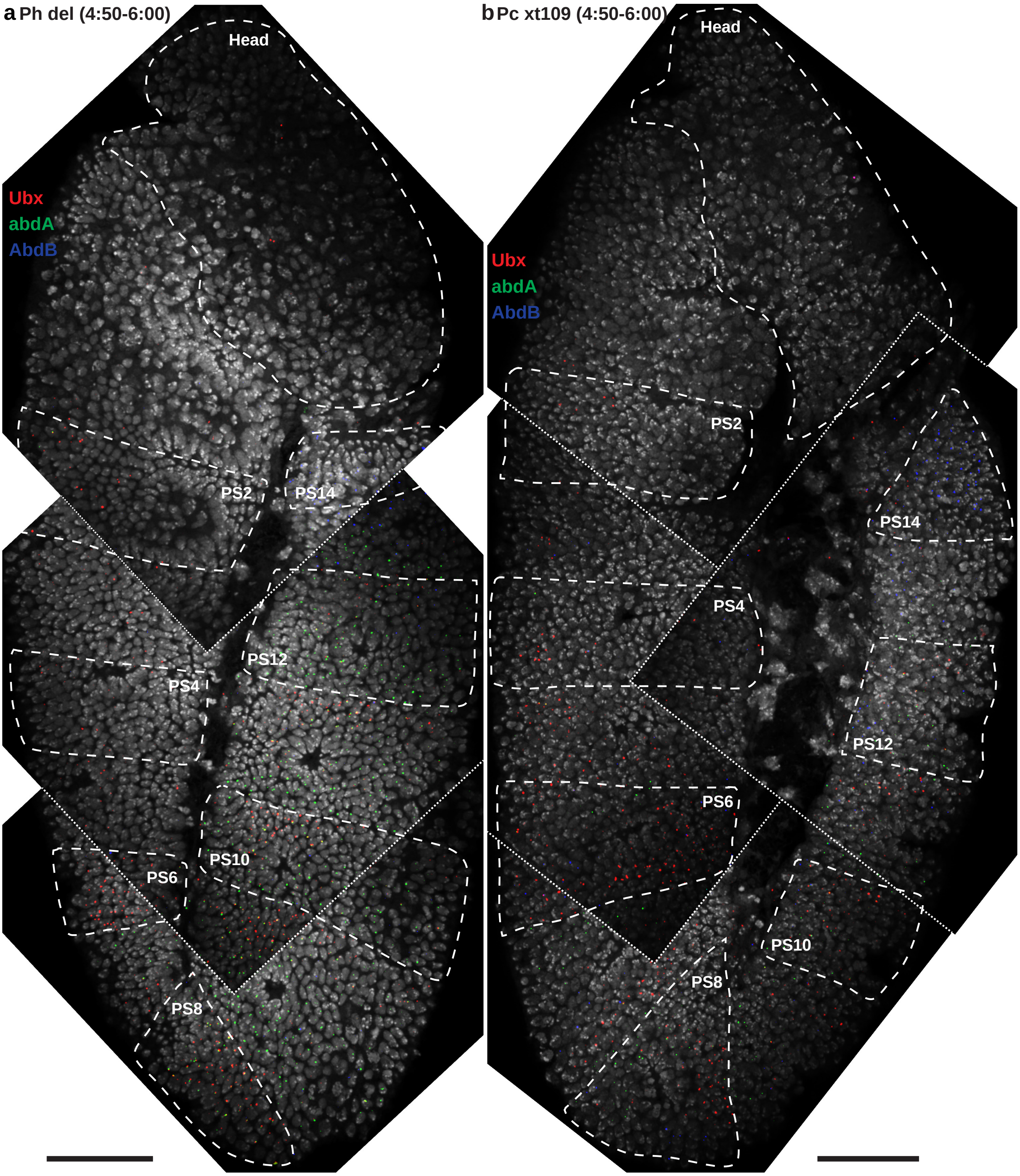

Supplementary Figure 3: Hox genes of BX-C derepression in Ph^{del} and Pc^{XT109} embryos 4:50-6:00 after fertilization. Images of RNA FISH experiments illustrating ectopic expression of *Ubx*, *adbA*, and *AbdB* occurring in *ph*^{del} (a) and *Pc*^{XT109} (b) embryos. These images are maximum intensity projections of confocal images and dash lines indicate where two images were merged. They illustrate the quantification of Hox gene derepression shown in Figure 2 and supplementary Figure 2. Scale bars, 50 µm.

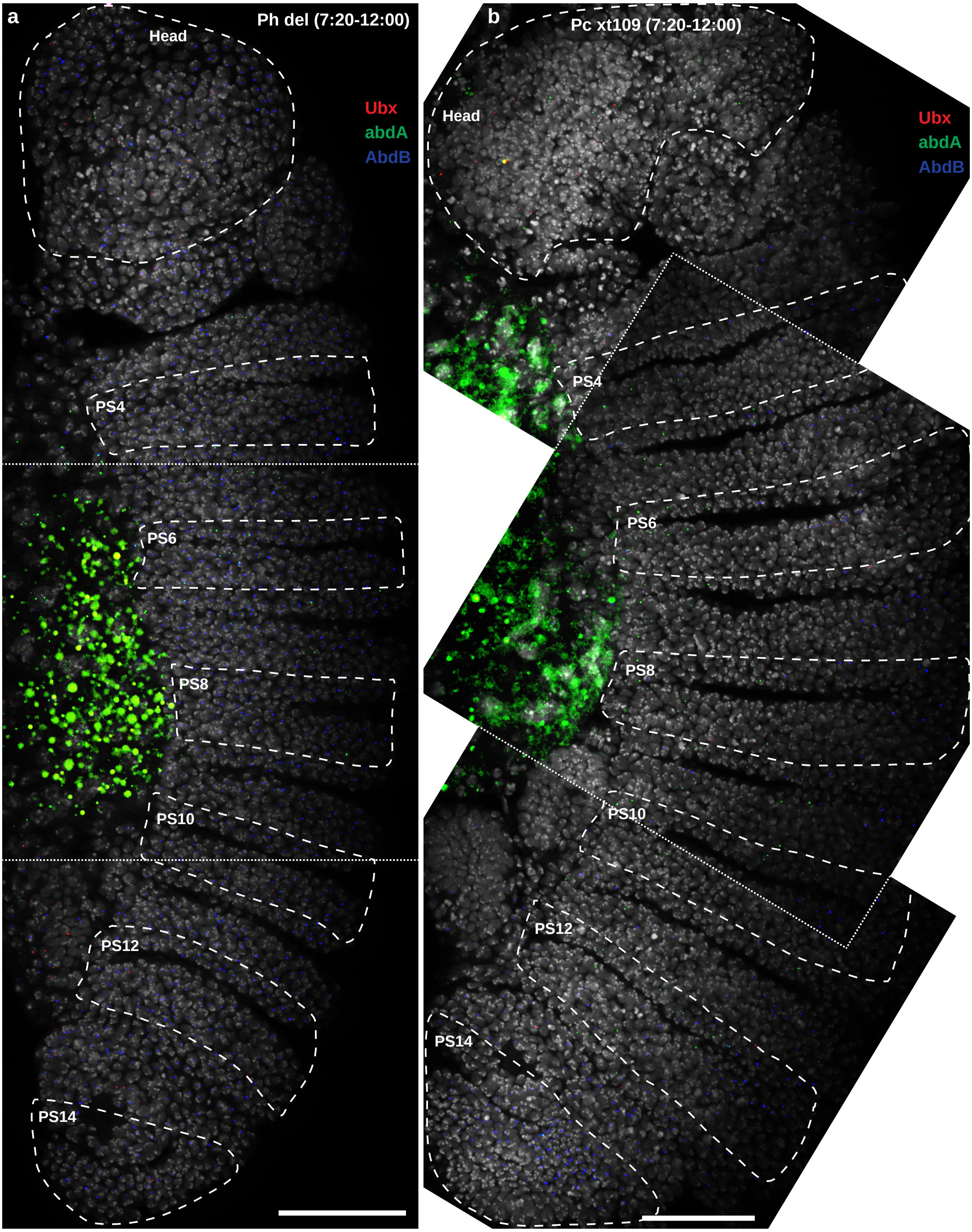

Supplementary Figure 4: Hox genes of BX-C derepression in Ph^{del} and Pc^{XT109} embryos 7:20-12:00 after fertilization. Images of RNA FISH experiments illustrating ectopic expression of *Ubx*, *adbA*, and *AbdB* occurring in *ph^{del}* (a) and *Pc^{XT109}* (b) embryos. These images are maximum intensity projections of confocal images and dash lines indicate where two images were merged. They illustrate the quantification of Hox gene derepression shown in Figure 2 and supplementary Figure 2. Scale bars, 50 µm.

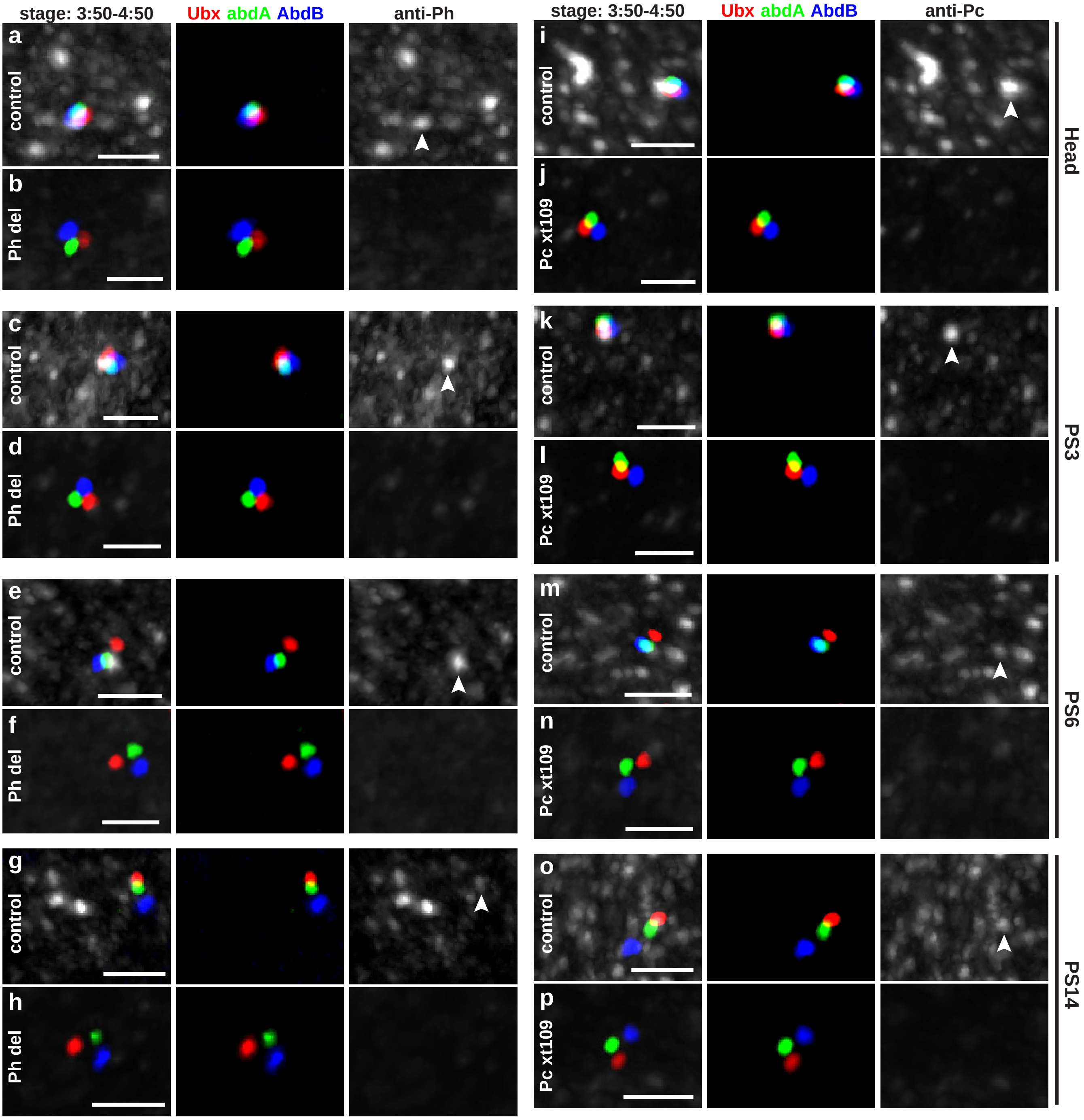

Supplementary Figure 5: Effect of Ph and Pc on BX-C folding in embryos 3:50-4:50 after fertilization. Images of immuno-DNA FISH experiments illustrating BX-C folding in embryos 3:50-4:50 after fertilization. An antibody anti-Ph was used to discriminate Ph^{del} mutant embryos (b, d, f, h) from control ones (a, c, e, g). An antibody anti-Pc was used to discriminate Pc^{XT109} mutant embryos (j, l, n, p) from control ones (i, k, m, o). In head and PS3, *Ubx*, *adbA* and *AbdB* are repressed and found inside a Ph/Pc foci (arrowhead) in control embryos (a, c, i, k), whereas BX-C folding is less compact in Ph^{del} (b, d) and Pc^{XT109} (j, l) embryos. *Ubx* is expressed in PS6 and located outside Ph/Pc foci in contrast to *abdA* and *AbdB* (arrowhead in e, m). The distance *abdA-AbdB* increases in Ph^{del} (f) and Pc^{XT109} (n) embryos. *AbdB* is expressed in PS14 and located outside Ph/Pc foci in contrast to *Ubx* and *abdA* (arrowhead in g, o). The distance *Ubx-abdA* increases in Ph^{del} (h) and Pc^{XT109} (p) embryos. Scale bars, 2 µm.

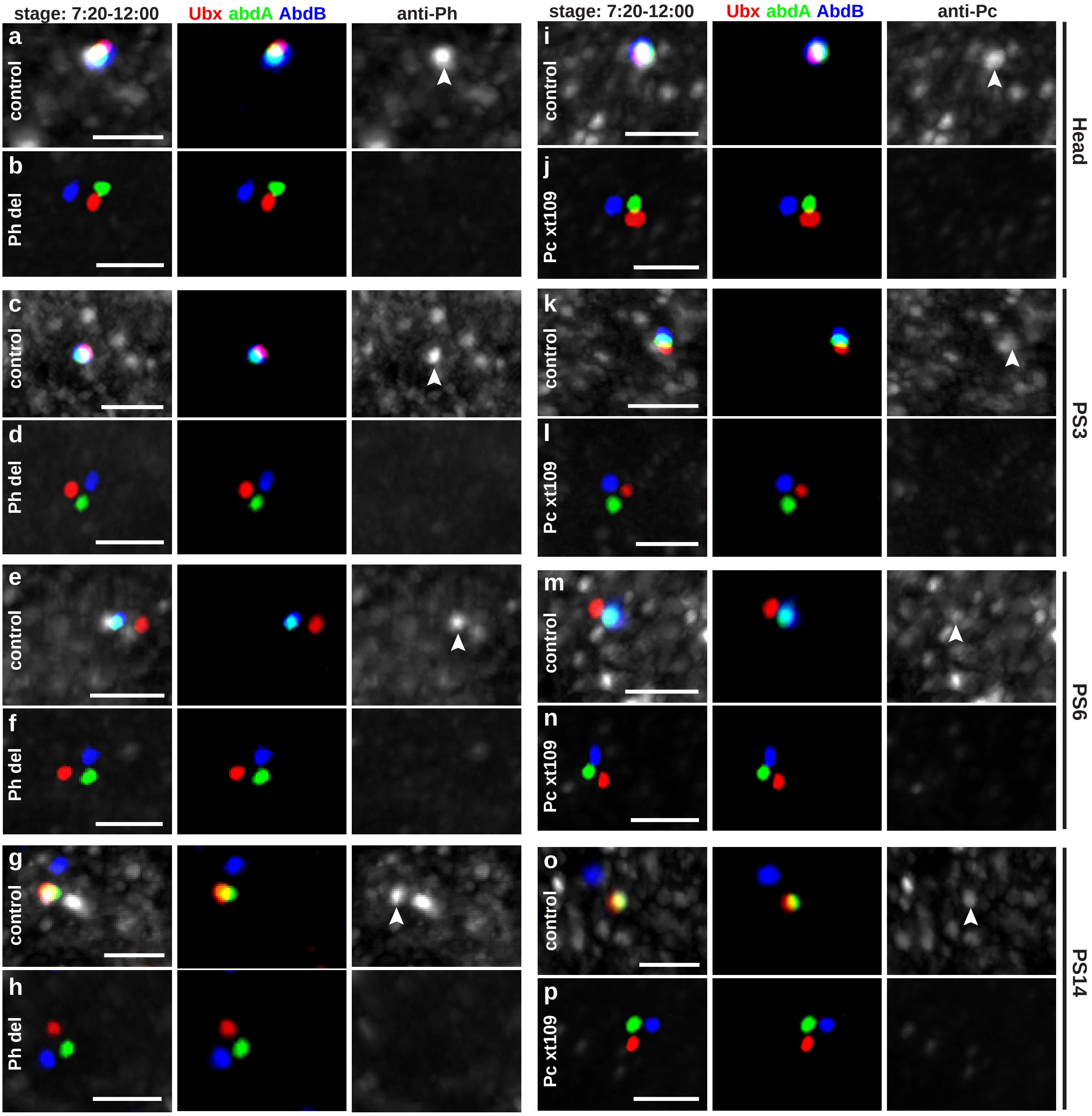

Supplementary Figure 6: Effect of Ph and Pc on BX-C folding in embryos 7:20-12:00 after fertilization. Images of immuno-DNA FISH experiments illustrating BX-C folding in embryos 7:20-12:00 after fertilization. An antibody anti-Ph was used to discriminate Ph^{del} mutant embryos (b, d, f, h) from control ones (a, c, e, g). An antibody anti-Pc was used to discriminate Pc^{XT109} mutant embryos (j, l, n, p) from control ones (i, k, m, o). In head and PS3, *Ubx*, *adbA* and *AbdB* are repressed and found inside a Ph/Pc foci (arrowhead) in control embryos (a, c, i, k), whereas BX-C folding is less compact in Ph^{del} (b, d) and Pc^{XT109} (j, l) embryos. *Ubx* is expressed in PS6 and located outside Ph/Pc foci in contrast to *abdA* and *AbdB* (arrowhead in e, m). The distance *abdA-AbdB* increases in Ph^{del} (f) and Pc^{XT109} (n) embryos. *AbdB* is expressed in PS14 and located outside Ph/Pc foci in contrast to *Ubx* and *abdA* (arrowhead in g, o). The distance *Ubx-abdA* increases in Ph^{del} (h) and Pc^{XT109} (p) embryos. Scale bars, 2 µm.

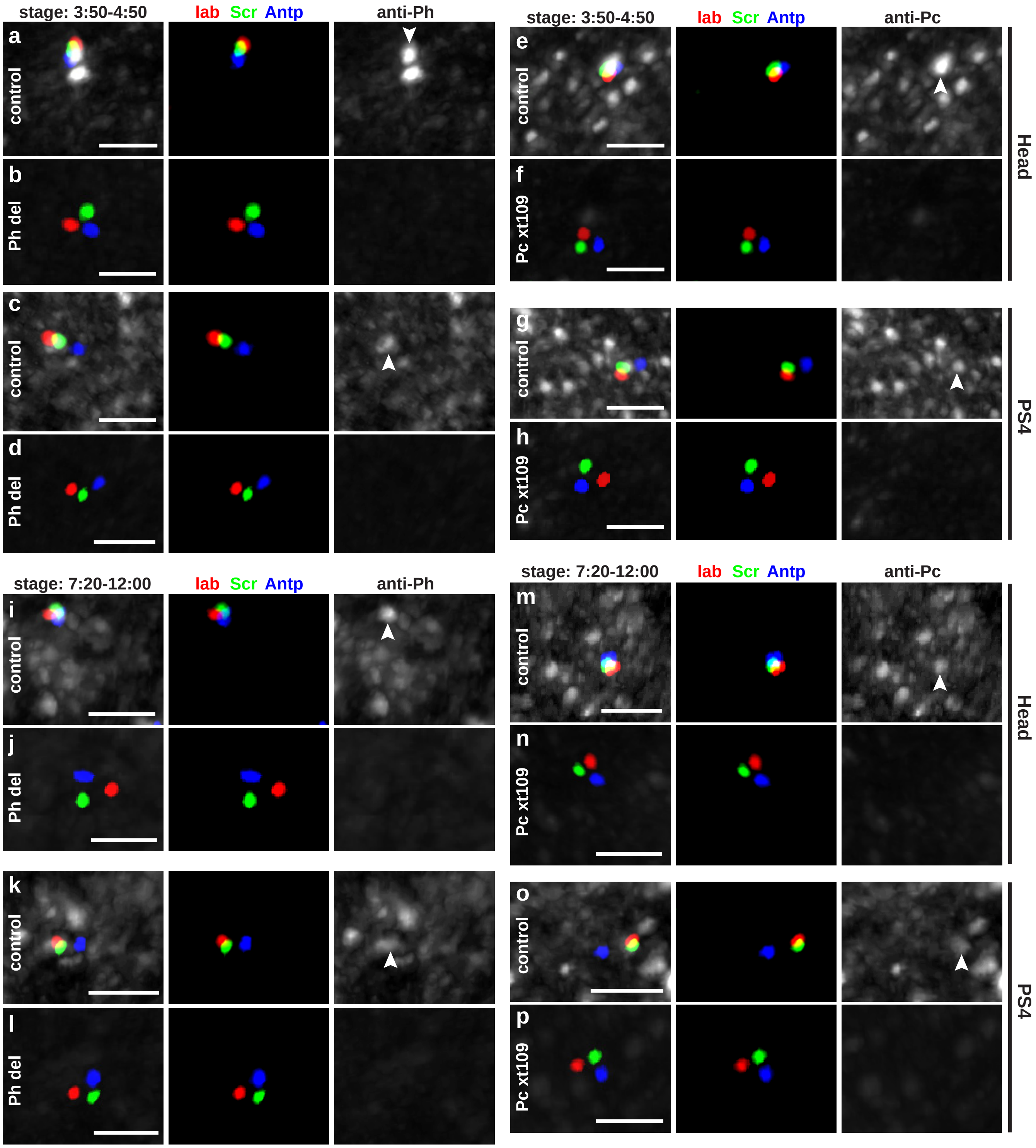

Supplementary Figure 7: Effect of Ph and Pc on ANT-C folding. Images of immuno-DNA FISH experiments illustrating ANT-C folding in embryos 3:50-4:50 (a-h) or 7:20-12:00 (i-p) after fertilization. An antibody anti-Ph was used to discriminate Ph^{del} mutant embryos (b, d, j, l) from control ones (a, c, i, k). An antibody anti-Pc was used to discriminate Pc^{XT109} mutant embryos (f, h, n, p) from control ones (e, g, m, o). In head, *lab*, *Scr* and *Antp* are repressed and found inside a Ph/Pc foci (arrowhead) in control embryos (a, e, i, m), whereas ANT-C folding is less compact in Ph^{del} (b, j) and Pc^{XT109} (f, n) embryos. *Antp* is expressed in PS4 and located outside Ph/Pc foci in contrast to *lab* and *Scr* (arrowhead in c, g, k, o). The distance *lab*-*Scr* increases in Ph^{del} (d, l) and Pc^{XT109} (h, p) embryos. Scale bars, 2 µm.

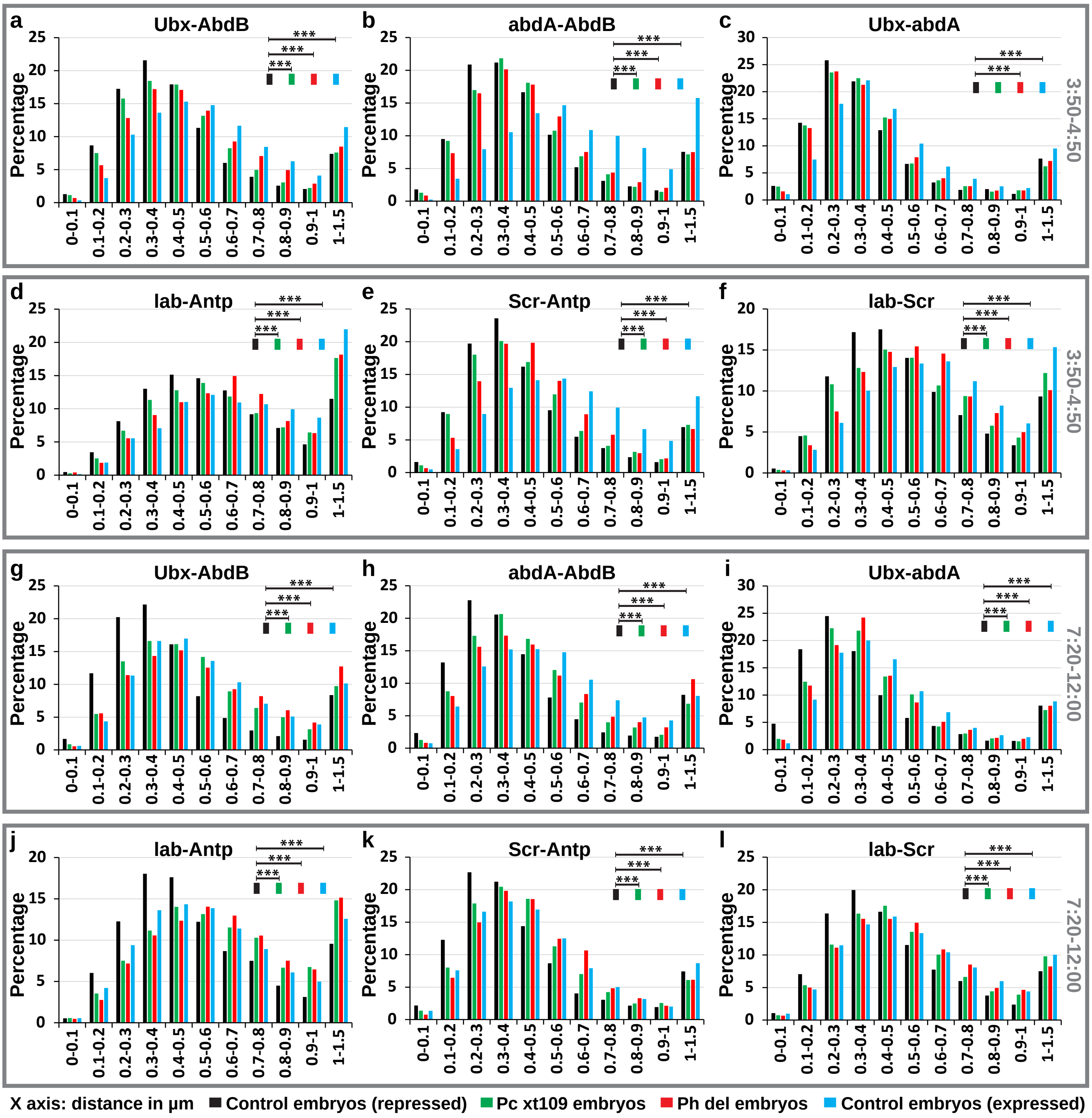

Supplementary Figure 8: Comparison between the effects of Ph, Pc and Hox gene transcription on BX-C and ANT-C folding. Histograms showing the distance distributions of *Ubx-AbdB* (a, g), abdA-*AbdB* (b, h), *Ubx-abdA* (c, i), *lab-Antp* (d, j), *Scr-Antp* (e, k), and *lab-Scr* (f-l) measured in the cell nuclei of embryos at the 3:50-4:50 (a-f) and 7:20-12:00 (g-l) stages after fertilization. To characterize the effects of Ph and Pc on the folding of completely repressed BX-C (corresponding to Fig. 3) and ANT-C (corresponding to Fig. 4), histograms show distances (in µm) measured within the head and PS0 (a-c; g-i) or the head (d-f; j-l) of ph^{del} (red), Pc^{XT109} (green) and control embryos (merge of ph^{del} and Pc^{XT109} control embryos in black). To compare the effects of Ph and Pc on BX-C and ANT-C folding with the transcription of Hox genes, histograms also present distances measured in the parasegment of control embryos wherein Hox genes are expressed (blue: PS6-8 for distances *Ubx-abdA* and *Ubx-AbdB* and PS14 for distance *abdA-AbdB*; PS4-PS5 for distances *lab-Antp* and *Scr-Antp* and PS1-PS2 for distance *lab-Scr*). Significant differences are indicated (Mann-Whitney, U test, two-tailed, * $P < 0.05$; ** $P < 0.01$; *** $P < 0.001$).

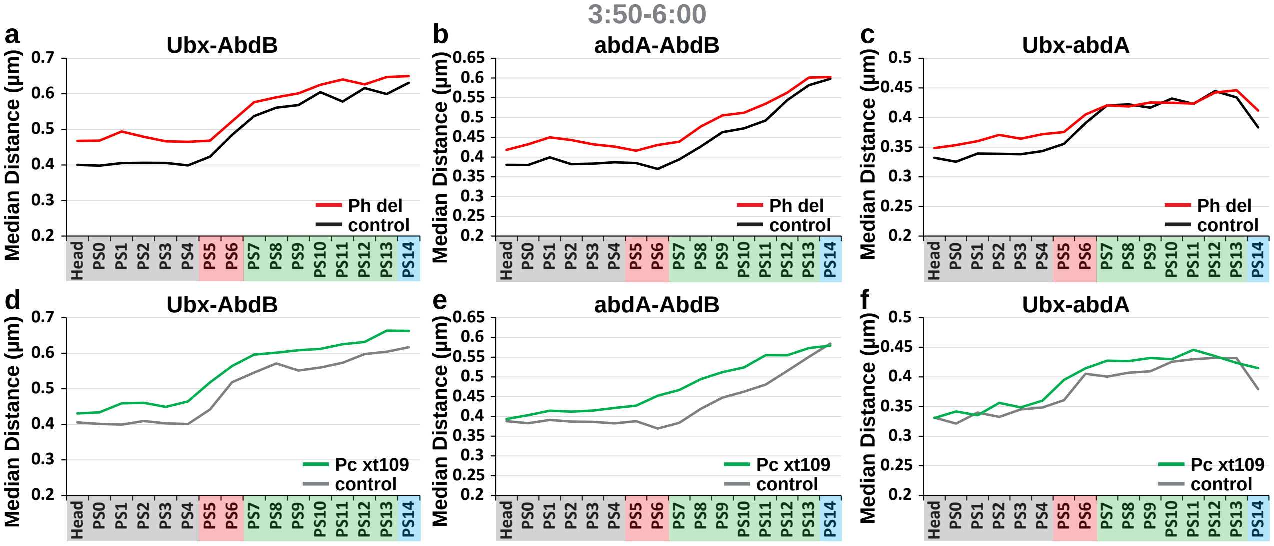

g

		Head	PS0	PS1	PS2	PS3	PS4	PS5	PS6	PS7	PS8	PS9	PS10	PS11	PS12	PS13	PS14
Ubx-AbdB	Ph del vs. control	2.2E-13	3.4E-09	4.4E-09	7.6E-06	7.4E-05	2.5E-06	4.5E-05	6.0E-03	1.0E-02	4.6E-02	3.4E-02	1.0E-01	4.1E-04	2.3E-01	2.0E-03	2.4E-01
	Pc xt109 vs. Control	7.3E-03	1.5E-03	2.0E-06	1.7E-03	1.9E-03	2.1E-05	7.3E-05	7.6E-03	2.9E-03	5.1E-02	4.7E-04	1.1E-02	1.6E-02	1.0E-02	2.8E-02	5.6E-02
abdA-AbdB	Ph del vs. control	1.1E-06	1.0E-07	3.9E-05	2.8E-05	3.9E-06	1.2E-03	2.2E-03	2.2E-05	2.5E-03	1.2E-03	2.3E-03	1.4E-03	1.6E-03	1.6E-01	9.5E-02	4.0E-01
	Pc xt109 vs. Control	2.6E-01	3.4E-02	6.6E-03	7.3E-03	1.9E-02	3.5E-03	6.4E-04	4.6E-06	1.0E-05	1.1E-04	1.7E-03	4.3E-03	4.4E-05	7.3E-03	6.4E-02	3.9E-01
Ubx-abdA	Ph del vs. control	1.5E-03	3.4E-04	4.6E-03	1.2E-03	6.8E-03	1.2E-02	3.2E-02	1.0E-01	5.0E-01	3.9E-01	2.3E-01	2.9E-01	4.9E-01	4.3E-01	1.5E-01	1.7E-02
	Pc xt109 vs. Control	4.6E-01	2.2E-02	2.9E-01	2.2E-02	3.8E-01	1.9E-01	8.3E-03	2.1E-01	2.1E-02	4.1E-02	2.5E-02	3.7E-01	1.5E-01	4.1E-01	3.0E-01	1.5E-02

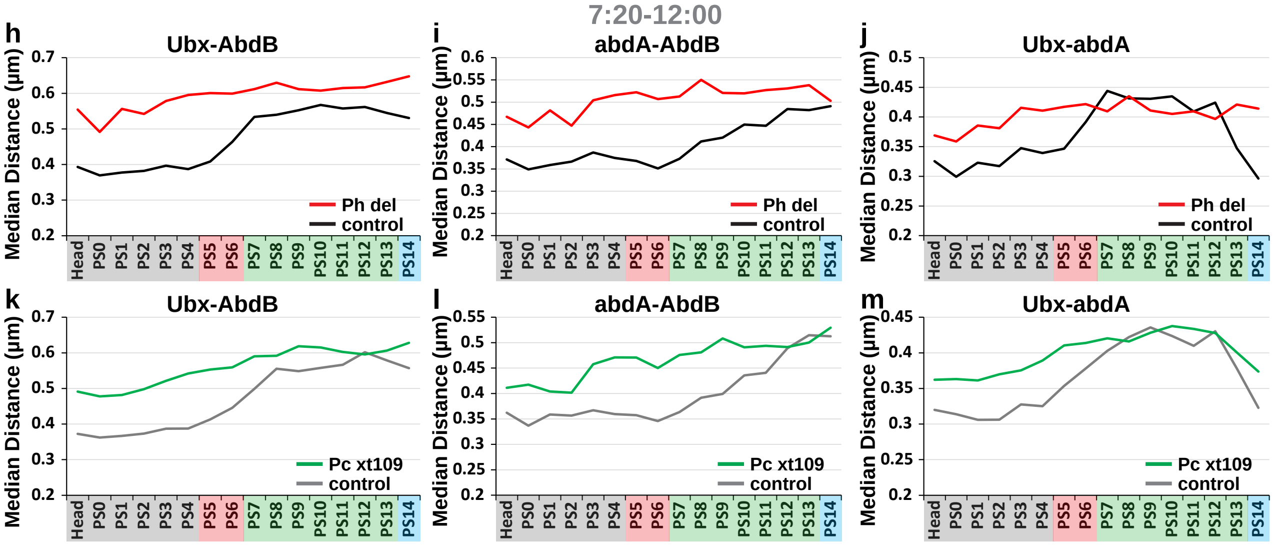

n

		Head	PS0	PS1	PS2	PS3	PS4	PS5	PS6	PS7	PS8	PS9	PS10	PS11	PS12	PS13	PS14
Ubx-AbdB	Ph del vs. control	6.4E-08	1.9E-04	6.0E-09	4.0E-04	1.4E-08	3.5E-08	3.2E-08	1.5E-06	8.4E-04	9.3E-04	5.5E-03	3.8E-02	9.1E-03	3.0E-03	1.4E-03	1.8E-02
	Pc xt109 vs. Control	7.7E-09	6.3E-05	3.4E-08	3.8E-06	2.4E-08	1.0E-10	3.7E-07	7.7E-07	3.7E-05	5.4E-02	1.3E-04	1.9E-03	2.1E-02	3.6E-01	1.2E-01	6.6E-02
abdA-AbdB	Ph del vs. control	2.5E-07	4.2E-03	7.4E-08	8.2E-03	5.4E-06	3.4E-07	1.8E-07	1.0E-08	2.0E-08	3.0E-06	2.9E-05	1.0E-04	2.0E-05	5.8E-03	9.7E-03	3.8E-01
	Pc xt109 vs. Control	6.5E-04	4.7E-04	5.2E-03	4.6E-04	2.2E-06	1.8E-06	6.8E-07	1.3E-07	2.5E-09	7.6E-07	2.0E-06	5.7E-03	5.5E-04	4.4E-01	2.7E-01	3.4E-01
Ubx-abdA	Ph del vs. control	7.7E-04	8.6E-04	3.8E-05	1.8E-02	1.6E-05	8.9E-07	1.9E-05	4.1E-03	6.6E-03	4.2E-01	1.5E-01	1.9E-02	4.9E-01	4.4E-02	2.3E-06	1.1E-04
	Pc xt109 vs. Control	6.2E-03	3.8E-03	5.3E-04	2.1E-05	4.9E-03	2.1E-04	9.1E-04	8.3E-03	1.5E-01	3.3E-01	3.3E-01	2.1E-01	5.1E-02	4.2E-01	1.5E-01	1.6E-02

Supplementary Figure 9: Effects of Ph and Pc on BX-C folding in early and late embryos. The distances *Ubx-AbdB* (a, d, h, k), *abdA-AbdB* (b, e, i, l), and *Ubx-abdA* (c, f, j, m) were measured in the cell nuclei of ph^{del} (red in a-c; h-j) embryos and their respective controls (black in a-c; h-j) or Pc^{XT109} (green in d-f; k-m) embryos and their respective controls (grey in d-f; k-m) at 3:50-6:00 (a-f) and 7:20-12:00 (h-m) after fertilization. The highlighted PSs show regions wherein none of *Ubx*, *abdA* and *AbdB* (grey), only *Ubx* (red), both *Ubx* and *abdA* (green) or only *AbdB* (blue) are expressed. For each PS of each embryo, we computed the median distances *Ubx-abdA*, *abdA-AbdB* and *Ubx-AbdB*. Curves show the mean of median distances from several embryos calculated for each PS along the anteroposterior axis. g and n: Tables of p values comparing the distances *Ubx-AbdB*, *abdA-AbdB* and *Ubx-abdA* measured in ph^{del} embryos with the those measured in their respective control embryos and in Pc^{XT109} embryos with the those measured in their respective controls along the anteroposterior axis at the 3:50-6:00 (g) and 7:20-12:00 (n) stages after fertilization (t-test, one-tailed, yellow: $P < 0.05$; orange: $P < 0.01$; red: $P < 0.001$).

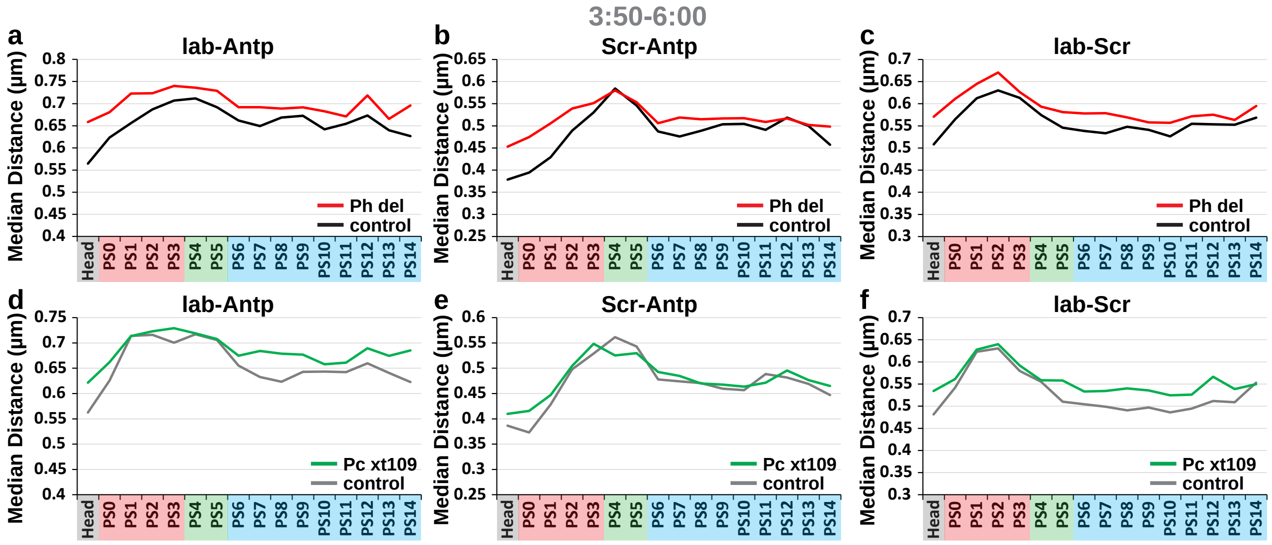

g

		Head	PS0	PS1	PS2	PS3	PS4	PS5	PS6	PS7	PS8	PS9	PS10	PS11	PS12	PS13	PS14
lab-Antp	Ph del vs. control	2.8E-09	3.2E-04	1.0E-04	1.5E-02	3.3E-02	6.7E-02	6.1E-02	1.2E-01	3.3E-02	2.3E-01	3.0E-01	8.6E-02	1.8E-01	6.1E-02	2.0E-01	7.2E-03
	Pc xt109 vs. Control	6.2E-04	5.3E-02	5.0E-01	3.2E-01	8.8E-02	4.7E-01	4.6E-01	1.9E-01	5.2E-03	4.8E-03	1.4E-01	3.3E-01	2.6E-01	1.7E-01	7.2E-02	1.7E-02
Scr-Antp	Ph del vs. control	1.7E-08	2.9E-06	3.1E-07	1.2E-03	1.4E-01	4.2E-01	3.6E-01	2.0E-01	3.2E-02	9.9E-02	2.8E-01	3.1E-01	1.9E-01	4.7E-01	4.6E-01	5.4E-02
	Pc xt109 vs. Control	1.1E-02	1.7E-03	8.4E-02	3.1E-01	1.1E-01	1.6E-02	2.0E-01	1.5E-01	2.3E-01	4.7E-01	3.6E-01	3.9E-01	2.5E-01	3.1E-01	3.9E-01	2.1E-01
lab-Scr	Ph del vs. control	4.9E-05	2.4E-03	4.5E-02	8.8E-03	2.4E-01	1.2E-01	2.5E-02	5.9E-02	1.4E-02	1.9E-01	2.7E-01	5.2E-02	1.8E-01	1.7E-01	3.4E-01	1.5E-01
	Pc xt109 vs. Control	3.9E-04	1.5E-01	4.0E-01	2.9E-01	2.1E-01	4.4E-01	2.8E-03	6.3E-02	3.0E-02	1.4E-02	7.9E-02	1.2E-01	1.5E-01	6.3E-02	1.4E-01	4.4E-01

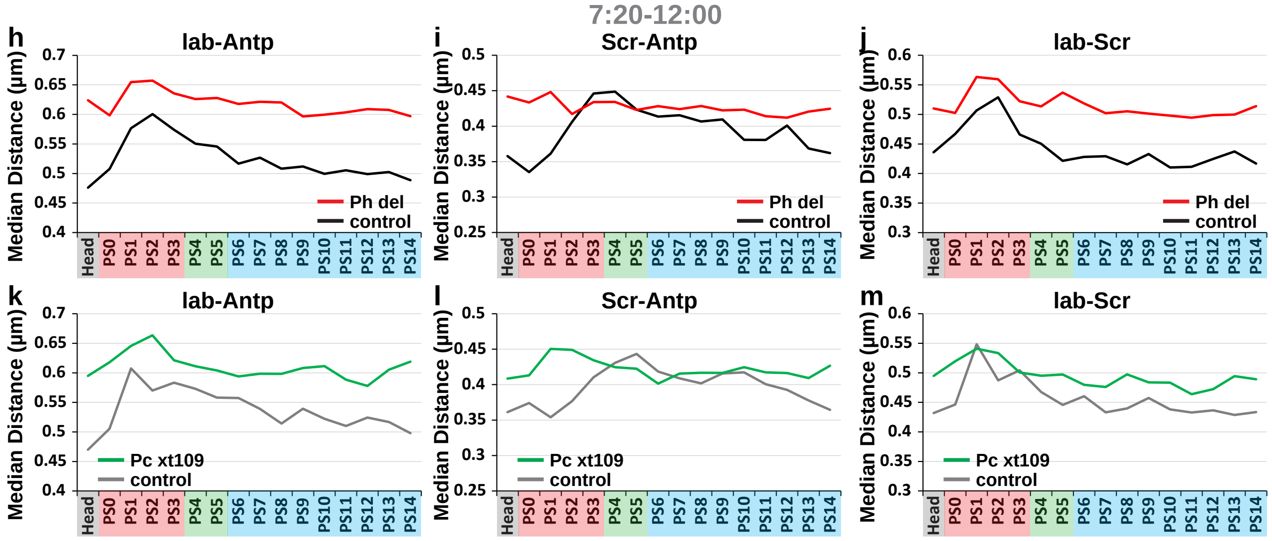

n

		Head	PS0	PS1	PS2	PS3	PS4	PS5	PS6	PS7	PS8	PS9	PS10	PS11	PS12	PS13	PS14
lab-Antp	Ph del vs. control	6.1E-09	6.7E-03	9.1E-04	4.4E-02	2.4E-03	6.0E-06	1.8E-05	4.4E-07	1.4E-05	2.3E-07	2.6E-06	1.9E-05	7.7E-06	6.4E-06	2.0E-06	9.1E-03
	Pc xt109 vs. Control	6.5E-07	3.3E-02	4.3E-02	4.9E-03	1.7E-02	3.6E-02	1.9E-02	3.2E-02	1.8E-03	2.4E-05	7.7E-05	4.3E-05	9.1E-04	8.1E-04	2.0E-03	3.9E-05
Scr-Antp	Ph del vs. control	1.4E-07	2.2E-06	1.7E-05	3.0E-01	1.8E-01	1.6E-01	4.9E-01	1.3E-01	2.2E-01	5.7E-02	2.1E-01	2.2E-03	1.9E-03	1.8E-01	1.9E-04	1.0E-03
	Pc xt109 vs. Control	5.0E-04	1.4E-01	2.3E-06	6.5E-05	4.1E-02	3.8E-01	1.3E-01	1.2E-01	3.3E-01	1.5E-01	4.8E-01	2.6E-01	1.3E-01	4.8E-02	3.9E-02	3.0E-03
lab-Scr	Ph del vs. control	2.0E-06	1.4E-01	3.0E-03	1.8E-01	1.9E-04	1.5E-04	5.9E-09	1.4E-06	1.3E-05	1.5E-07	4.5E-04	1.2E-07	2.4E-05	6.7E-05	4.6E-04	3.7E-03
	Pc xt109 vs. Control	1.2E-03	6.0E-02	3.7E-01	4.3E-02	4.1E-01	5.3E-02	6.7E-03	1.4E-01	7.6E-03	5.4E-04	1.2E-01	1.3E-03	4.1E-02	2.5E-02	8.3E-04	5.7E-03

Supplementary Figure 10: Effects of Ph and Pc on ANT-C folding in early and late embryos. The distances *lab-Antp* (a, d, h, k), *Scr-Antp* (b, e, i, l), and *lab-Scr* (c, f, j, m) were measured in the cell nuclei of ph^{del} (red in a-c; h-j) embryos and their respective controls (black in a-c; h-j) and in Pc^{XT109} (green in d-f; k-m) embryos and their respective controls (grey in d-f; k-m) at the 3:50-6:00 (a-f) and 7:20-12:00 (h-m) stages after fertilization. The highlighted PSs show regions wherein *lab*, *Scr* and *Antp* are repressed (grey), Hox genes located between *lab* and *Scr* are expressed (red), and where *Antp* is fully (green) or mildly (blue) expressed. For each PS of each embryo, we computed the median distances *lab-Scr*, *Scr-Antp* and *lab-Antp*. Curves show the mean median distances from several embryos calculated for each PS along the anteroposterior axis. g and n: Tables of p values comparing the distances *lab-Antp*, *Scr-Antp* and *lab-Scr* measured in ph^{del} embryos with the those measured in their respective controls and in Pc^{XT109} embryos with the those measured in their respective controls along the anteroposterior axis at the 3:50-6:00 (g) and 7:20-12:00 (n) stages after fertilization (t-test, one-tailed, yellow: $P < 0.05$; orange: $P < 0.01$; red: $P < 0.001$).

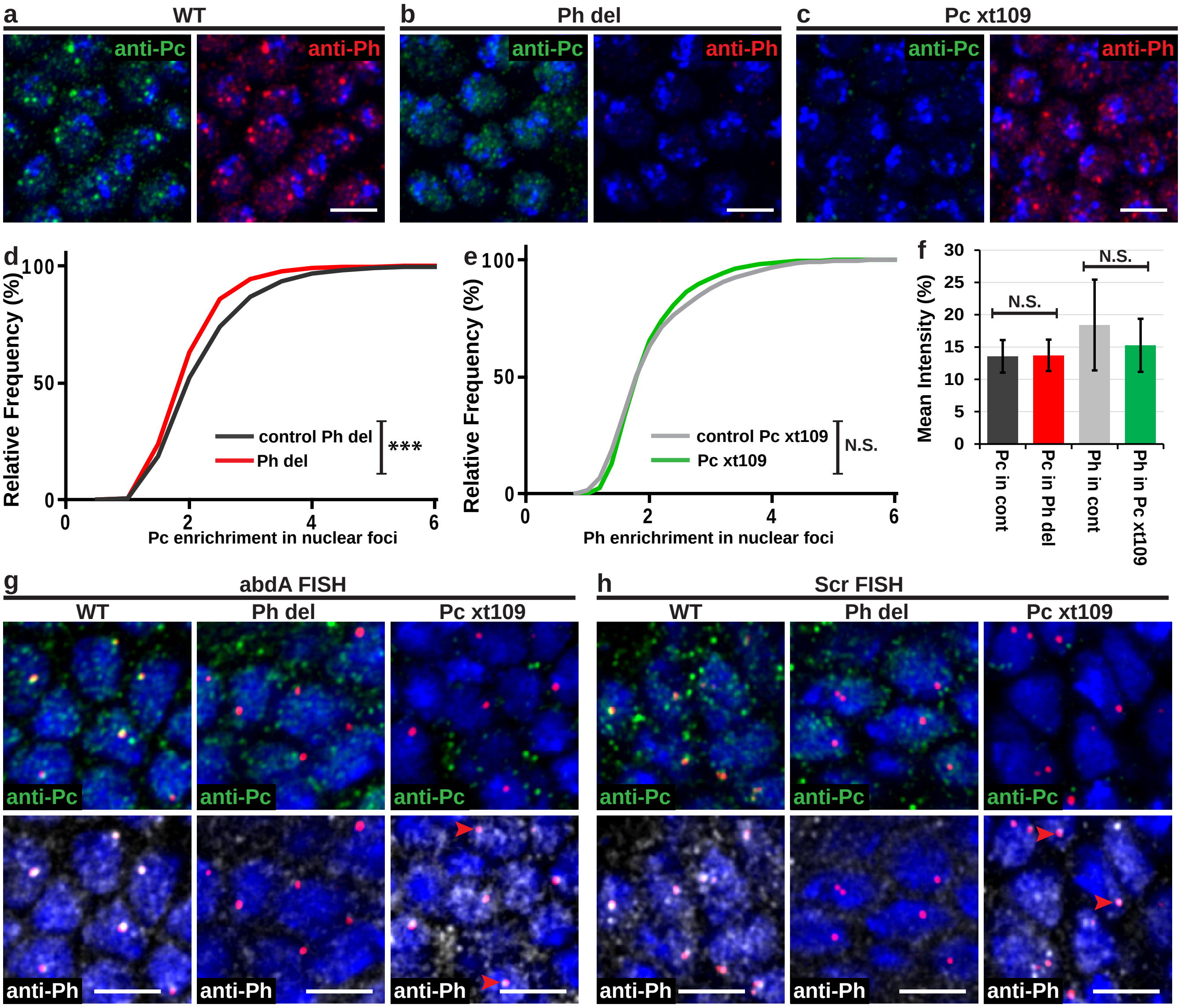

Supplementary Figure 11: Formation of Ph foci in the absence of Pc within embryos 3:50-7:20 after fertilization. a-c, Images of immunolabelling experiments performed in WT (a), ph^{del} (b) and Pc^{XT109} (c) embryos showing the distribution of Pc (green) and Ph (red) inside cell nuclei. d, e, cumulative histograms comparing the enrichment of Pc in nuclear foci between Ph^{del} and their controls embryos (d), or the enrichment of Ph in nuclear foci between $Pc^{XT109}$and their control embryos (e) (Mann-Whitney, U test, two-tailed, *** $P < 0.001$). f, chart comparing the mean nuclear intensity with errors bars (SD; N≥7) of Pc in Ph^{del} and their control embryos, and Ph in $Pc^{XT109}$and their control embryos (t-test, one-tailed). g, h, Pictures of immuno-DNA FISH experiments using anti-Pc (green), anti-Ph (grey) and DNA probes (red) recognizing the promoters of *abdA* (g) or *Scr* (h) performed in WT, ph^{del} and Pc^{XT109} embryos. Scale bars, 5 µm. Ph can form nuclear foci containing *abdA* or *Scr* (red arrows) in the absence of Pc, whereas the reverse is not true.

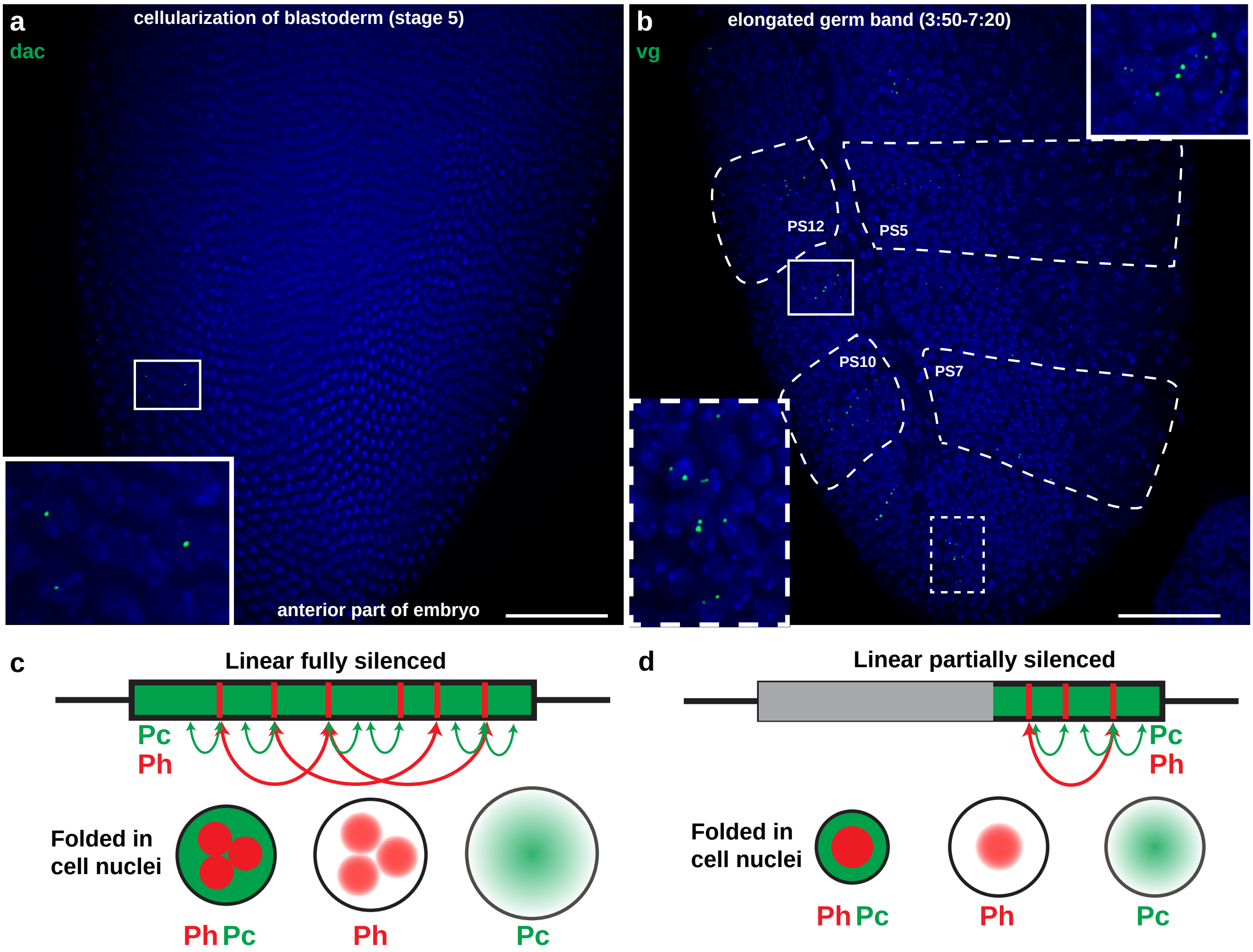

Supplementary Figure 12: RNA FISH can detect early transcription of dac and vg and model depicting the effect of Ph and Pc on Hox clusters folding. a, b, Images of RNA FISH experiments showing dac expression in embryos at developmental stage 5 (a) and vg expression in embryos at elongated germ band stage (b). Scale bars, 50 µm. c, d, Model depicting a cautious interpretation of the results. The top part shows a linear representation of totally (c) or partially (d) repressed Hox domains and the bottom part represents schematically their folding inside the cell nucleus. They are composed of discrete sites bound by Ph and Pc and wide regions containing H3K27me3 where Pc can bind. In WT cells, Ph binds discrete sites and might generate loops (red arrows) through its ability to oligomerize. On the other hand, Pc might further compact repressed chromatin domains by local interactions between discrete sites and regions containing H3K27me3 (green arrows). These two distinct forces would compact large repressed chromatin domains and form PRC1 nuclear foci. In the absence of Pc, Ph might still produce loops between discrete sites and fold repressed chromatin domains, therefore forming nuclear foci. The compaction effect of Ph in the absence of Pc would be weaker for partially repressed chromatin domains because fewer loops involving a smaller linear domain would exist. In contrast, no loops might be established in the absence of Ph, and the local effect of Pc might not be sufficient to form nuclear foci, explaining the stronger effect of *Ph* mutations than that of *Pc* mutations.

Supplementary Note 1

Statistical information

We listed the numbers of measurements made in each figure of this study. The numbers of embryos in which we measured the density of RNA FISH spots are shown in Supplementary Tables 2, 5-7. For DNA FISH experiments, the numbers of distances measured and the numbers of embryos for each condition are listed in Supplementary Tables 3-4, 8-9, 11-12. We listed in Supplementary Tables 10 and 13, the numbers of Pc/Ph foci and the numbers of embryos used to quantify Pc and Ph enrichments.

Fig. 1

RNA FISH: Number (No.) of embryos in Supplementary Table 2

DNA FISH (No. of distances analysed) in Supplementary Table 3-4

Fig. 2 and Supplementary Fig. 2

RNA FISH (No. of embryos) in Supplementary Table 5-7

Fig. 3-5

DNA FISH: BX-C in Supplementary Table 8

DNA FISH: ANT-C in Supplementary Table 9

Fig. 5g, h

Quantification of Pc/Ph enrichments in Supplementary Table 10

Supplementary Fig. 8

DNA FISH in Supplementary Table 11

Supplementary Fig. 9-10

DNA FISH in Supplementary Table 12

Supplementary Fig. 11

Quantification of Pc/Ph enrichments in Supplementary Table 13

Supplementary Table 1: List of primers used in this study

primers name	sens (sequence)	anti-sens (sequence)	experiments
Ubx	ggcaatggtgtttctttggt	tggctgtgatctcgcttaga	RNA FISH
abdA	gcggatcgcgtaaattaaac	tcggatctgaatgtgagtgc	RNA FISH
AbdB	catgcgagtagaaagccaca	cggaagttaaagacggcaat	RNA FISH
lab	tgagccataaacacctgtgc	ggcaattaagaaaccgcatt	RNA FISH
pb	gtcgacggacggacatttac	ggcgatgttgtagcacagaa	RNA FISH
Dfd	tgcaacttggacatggagaa	acatcaaatgacacggacca	RNA FISH
Scr	atcctggtcttgccactgtt	acaagcgccgcatatagaag	RNA FISH
Antp	cattcagcggcacatcatta	tgatttctctggaggcttcg	RNA FISH
Ubx + T7	gtaatacgactcactatagggcggcaatggtgtttctttggt	gtaatacgactcactatagggctggctgtgatctcgcttaga	RNA FISH
abdA + T7	gtaatacgactcactatagggcgcggatcgcgtaaattaaac	gtaatacgactcactatagggctcggatctgaatgtgagtgc	RNA FISH
AbdB + T7	gtaatacgactcactatagggccatgcgagtagaaagccaca	gtaatacgactcactatagggccggaagttaaagacggcaat	RNA FISH
lab + T7	gtaatacgactcactatagggctgagccataaacacctgtgc	gtaatacgactcactatagggcggcaattaagaaaccgcatt	RNA FISH
pb + T7	gtaatacgactcactatagggcgtcgacggacggacatttac	gtaatacgactcactatagggcggcgatgttgtagcacagaa	RNA FISH
Dfd + T7	gtaatacgactcactatagggctgcaacttggacatggagaa	gtaatacgactcactatagggcacatcaaatgacacggacca	RNA FISH
Scr + T7	gtaatacgactcactatagggcatcctggtcttgccactgtt	gtaatacgactcactatagggcacaagcgccgcatatagaag	RNA FISH
Antp + T7	gtaatacgactcactatagggccattcagcggcacatcatta	gtaatacgactcactatagggctgatttctctggaggcttcg	RNA FISH
Antp	cgaaatccgaaaaatctgga	gcagaaatcatacggccatt	DNA FISH
	ggacccgatgaagtcgataa	ggcagccatttctctctcag	DNA FISH
	tataccttgcccactgcaca	cacaacaaagcactcgagga	DNA FISH
	cgctgagctgcaaactgtag	gagagcgagagcgtaggaga	DNA FISH
	ggtacactccccttcagcaa	tggcgtacaaacaccactgt	DNA FISH
	ccgaaaatatcgatggagga	tgacgggcatttatcaaaca	DNA FISH
Scr	atcagcgtcttggctgactt	tgatgacgatgacggagtgt	DNA FISH
	aattttcagcttgcgtgctt	ttaagcgctttggaacaggt	DNA FISH
	tggctagacttttgcgtcct	cactgtggtgtggaggattg	DNA FISH
	ccacgttcaattcacgtttg	ggccaaccataaagagctga	DNA FISH
	aaaggagcagtaaggcacga	acacgcatagtgtgggtgaa	DNA FISH
	gggaaagacggattgcagta	agccattgacccaaattcag	DNA FISH
lab	gcattcctggtggtcacttt	ctcccattgccatgaagatt	DNA FISH
	cgtctcccagcatccagtat	gcctgtgcagagtcagtcag	DNA FISH
	tctacaactcgcgtcattcg	ctatccgcattcgcatctct	DNA FISH
	ctgcaaatgctgatgctgtt	tttccaatcccatagcaagc	DNA FISH
	agaggcgacaagcctctaca	tgttgcagtgagaagcgact	DNA FISH
	taaagccaaccggcattaag	gcgcagtctgtacgtggtta	DNA FISH
Ubx	gaccgccaaggtgcttatta	tcgccgatttgatttactcc	DNA FISH
	cgcaaggctctcgaatttag	gaaagcaggcagaacagacc	DNA FISH
	cccgtgttccaaatgtcttt	taggccgagtcgagtgagtt	DNA FISH
	cccgtatttgctcggtttta	ccaactttctgcaggaggag	DNA FISH
	tcatcggtagcttgttgcag	tggtccatttcgttcactca	DNA FISH
	acattgcgacagtgtcaagc	tgtttgtcctggcattggta	DNA FISH
abdA	ttgacacccaaatctggtca	cctggatgacgcttacaggt	DNA FISH
	attgttggtgatccctgctc	ccacagttccagccgtttat	DNA FISH
	gaattcagcgtggagaggag	gcggcaatacgattcagttt	DNA FISH
	ttgctctcatcctccttcgt	tgggcgttttacatttttcc	DNA FISH
	tcccagggataagtgtcgag	acacacacagccagacaagc	DNA FISH
	caagtggtgttgtgggacag	gctgcatattggtgacatgg	DNA FISH
AbdB	aagatgagcaggcgaacact	cagaccgagaaaacgagagg	DNA FISH
	aaggggtgcagttgtgaaag	atcacgtacggccataaagc	DNA FISH
	aaactcttttacggcggaca	caggaaccgaaatcgaagag	DNA FISH
	gctgccagataaaggacgac	gcaaatcgggctaaatgtgt	DNA FISH
	ggtctaatccggtgctgtgt	aatcggaacgggaaactctt	DNA FISH
	ttgattcccggactcttgac	acctgctgccaaagaaaatg	DNA FISH

Supplementary Table 2: Number of embryos analysed in Figure 1c

	Wi1118	Head	PS0	PS1	PS2	PS3	PS4	PS5	PS6	PS7	PS8	PS9	PS10	PS11	PS12	PS13	PS14
3:50-7:20	lab	25	25	25	25	25	25	25	25	25	25	19	19	19	19	19	19
	pb	24	24	24	24	24	24	24	24	24	24	18	18	18	18	18	18
	Dfd	47	47	47	47	47	47	47	47	47	45	38	37	37	37	37	37
	Scr	32	32	32	32	32	32	32	32	32	30	29	28	28	28	28	28
	Antp	43	43	43	43	43	43	43	43	43	41	40	39	39	39	39	39
	Ubx	28	24	24	24	24	24	24	24	24	25	23	23	23	23	23	23
	abdA	28	24	24	24	24	24	24	24	24	25	23	23	23	23	23	23
	AbdB	28	24	24	24	24	24	24	24	24	25	23	23	23	23	23	23

Supplementary Table 3: Number of distances measured in Figure 1d, h

	Head	PS0	PS1	PS2	PS3	PS4	PS5	PS6	PS7	PS8	PS9	PS10	PS11	PS12	PS13	PS14
Ubx-AbdB	7386	5168	4604	4776	5890	5221	4091	3522	3700	2842	3056	2909	3230	3465	3507	4053
abdA-AbdB	7386	5161	4602	4757	5902	5234	4103	3526	3730	2838	3004	2867	3182	3330	3498	4065
Ubx-abdA	7426	5169	4646	4805	5930	5253	4124	3548	3686	2838	3004	2862	3180	3367	3503	4032
Ubx-Pc	3713	2588	2324	2412	2959	2620	2056	1772	1828	1421	1528	1452	1614	1751	1756	2010
abdA-Pc	3713	2581	2322	2393	2971	2633	2068	1776	1858	1417	1476	1410	1566	1616	1747	2022
AbdB-Pc	3673	2580	2280	2364	2931	2601	2035	1750	1872	1421	1528	1457	1616	1714	1751	2043
No. of embryos	19	19	18	18	18	18	18	18	18	17	16	16	16	16	16	16

Supplementary Table 4: Number of distances measured in Figure 1e, i

	Head	PS0	PS1	PS2	PS3	PS4	PS5	PS6	PS7	PS8	PS9	PS10	PS11	PS12	PS13	PS14
lab-Antp	12311	4014	5330	4481	6620	5800	5136	4395	5547	4214	4425	5394	5713	5415	5659	5295
Scr-Antp	12335	4005	5282	4440	6607	5783	5105	4365	5500	4226	4413	5365	5695	5396	5663	5299
lab-Scr	12492	4071	5408	4579	6701	5961	5257	4482	5643	4314	4484	5455	5796	5497	5746	5402
lab-Pc	6234	2040	2728	2310	3357	2989	2644	2256	2845	2151	2248	2742	2907	2758	2871	2699
Scr-Pc	6258	2031	2680	2269	3344	2972	2613	2226	2798	2163	2236	2713	2889	2739	2875	2703
Antp-Pc	6077	1974	2602	2171	3263	2811	2492	2139	2702	2063	2177	2652	2806	2657	2788	2596
No. of embryos	19	19	17	17	17	17	17	17	17	17	15	16	16	16	16	16

Supplementary Table 5: Number of Wi 1118 embryos analysed in Figure 2 and Supplementary Figure 2

	Wi1118	Head	PS0	PS1	PS2	PS3	PS4	PS5	PS6	PS7	PS8	PS9	PS10	PS11	PS12	PS13	PS14
3:50-4:50	lab	7	7	7	7	7	7	7	7	7	7	5	5	5	5	5	5
	pb	7	7	7	7	7	7	7	7	7	7	5	5	5	5	5	5
	Dfd	17	17	17	17	17	17	17	17	17	17	14	13	13	13	13	13
	Scr	12	12	12	12	12	12	12	12	12	12	11	10	10	10	10	10
	Antp	14	14	14	14	14	14	14	14	14	14	13	12	12	12	12	12
	Ubx	8	6	6	6	6	6	6	6	6	7	6	6	6	6	6	6
	abdA	8	6	6	6	6	6	6	6	6	7	6	6	6	6	6	6
	AbdB	8	6	6	6	6	6	6	6	6	7	6	6	6	6	6	6
4:50-6:00	lab	11	11	11	11	11	11	11	11	11	11	7	7	7	7	7	7
	pb	10	10	10	10	10	10	10	10	10	10	6	6	6	6	6	6
	Dfd	18	18	18	18	18	18	18	18	18	16	12	12	12	12	12	12
	Scr	12	12	12	12	12	12	12	12	12	10	10	10	10	10	10	10
	Antp	18	18	18	18	18	18	18	18	18	16	16	16	16	16	16	16
	Ubx	11	11	11	11	11	11	11	11	11	11	9	9	9	9	9	9
	abdA	11	11	11	11	11	11	11	11	11	11	9	9	9	9	9	9
	AbdB	11	11	11	11	11	11	11	11	11	11	9	9	9	9	9	9
6:00-7:20	lab	7	7	7	7	7	7	7	7	7	7	7	7	7	7	7	7
	pb	7	7	7	7	7	7	7	7	7	7	7	7	7	7	7	7
	Dfd	12	12	12	12	12	12	12	12	12	12	12	12	12	12	12	12
	Scr	8	8	8	8	8	8	8	8	8	8	8	8	8	8	8	8
	Antp	11	11	11	11	11	11	11	11	11	11	11	11	11	11	11	11
	Ubx	9	7	7	7	7	7	7	7	7	7	8	8	8	8	8	8
	abdA	9	7	7	7	7	7	7	7	7	7	8	8	8	8	8	8
	AbdB	9	7	7	7	7	7	7	7	7	7	8	8	8	8	8	8
7:20-12:00	lab	21	22	17	21	22	22	22	22	22	22	22	21	21	21	21	20
	pb	21	22	17	21	22	22	22	22	22	22	22	21	21	21	21	20
	Dfd	27	20	27	26	28	28	28	28	28	28	28	28	28	28	28	25
	Scr	18	11	18	17	19	19	19	19	19	19	19	19	19	19	19	17
	Antp	30	24	26	29	32	32	32	32	32	32	32	31	31	31	31	29
	Ubx	21	21	21	21	21	21	21	21	21	21	21	21	21	21	21	21
	abdA	21	21	21	21	21	21	21	21	21	21	21	21	21	21	21	21
	AbdB	21	21	21	21	21	21	21	21	21	21	21	21	21	21	21	21

Supplementary Table 6: Number of Ph^{del} embryos analysed in Figure 2 and Supplementary Figure 2

	Ph del	Head	PS0	PS1	PS2	PS3	PS4	PS5	PS6	PS7	PS8	PS9	PS10	PS11	PS12	PS13	PS14
3:50-4:50	lab	5	5	5	5	5	5	5	5	5	5	5	5	5	5	5	5
	pb	5	5	5	5	5	5	5	5	5	5	5	5	5	5	5	5
	Dfd	6	6	6	6	6	6	6	6	6	6	6	6	6	6	6	6
	Scr	7	7	7	7	7	7	7	7	7	7	7	7	7	7	7	7
	Antp	11	11	11	11	11	11	11	11	11	11	11	11	11	11	11	11
	Ubx	5	5	5	5	5	5	5	5	5	5	5	5	5	5	5	4
	abdA	5	5	5	5	5	5	5	5	5	5	5	5	5	5	5	4
	AbdB	5	5	5	5	5	5	5	5	5	5	5	5	5	5	5	4
4:50-6:00	lab	7	8	8	8	8	8	8	8	8	8	7	7	7	7	7	7
	pb	9	9	9	9	9	9	9	9	9	9	8	8	8	8	8	8
	Dfd	13	14	14	14	14	14	14	14	14	13	10	10	10	10	10	10
	Scr	14	14	14	14	14	14	14	14	14	13	9	9	9	9	9	9
	Antp	22	23	23	23	23	23	23	23	23	22	17	17	17	17	17	17
	Ubx	16	15	15	15	15	15	15	15	15	12	9	10	10	10	10	10
	abdA	16	15	15	15	15	15	15	15	15	12	9	10	10	10	10	10
	AbdB	16	15	15	15	15	15	15	15	15	12	9	10	10	10	10	10
6:00-7:20	lab	6	6	6	6	6	6	6	6	6	6	6	6	6	6	6	6
	pb	8	10	10	10	10	10	10	10	10	10	10	10	10	10	10	10
	Dfd	8	8	8	8	8	8	8	8	8	7	7	7	7	7	7	7
	Scr	10	12	12	12	12	12	12	12	12	11	11	11	11	11	11	11
	Antp	16	18	18	18	18	18	18	18	18	17	17	17	17	17	17	17
	Ubx	9	7	7	7	7	7	7	7	7	7	7	8	8	8	8	8
	abdA	9	7	7	7	7	7	7	7	7	7	7	8	8	8	8	8
	AbdB	9	7	7	7	7	7	7	7	7	7	7	8	8	8	8	8
7:20-12:00	lab	10	13	14	14	14	14	14	14	14	14	14	14	14	14	14	12
	pb	10	13	14	14	14	14	14	14	14	14	14	14	14	14	14	12
	Dfd	12	12	12	12	12	12	12	12	12	12	12	12	12	12	11	9
	Scr	12	12	12	12	12	12	12	12	12	12	12	12	12	12	11	9
	Antp	22	25	26	26	26	26	26	26	26	26	26	26	26	26	25	21
	Ubx	18	12	15	17	18	18	18	18	18	18	17	17	17	16	15	13
	abdA	18	12	15	17	18	18	18	18	18	18	17	17	17	16	15	13
	AbdB	18	12	15	17	18	18	18	18	18	18	17	17	17	16	15	13

Supplementary Table 7: Number of Pc^{XT109} embryos analysed in Figure 2 and Supplementary Figure 2

	Pc xt109	Head	PS0	PS1	PS2	PS3	PS4	PS5	PS6	PS7	PS8	PS9	PS10	PS11	PS12	PS13	PS14
3:50-4:50	lab	ND	ND	ND	ND	ND	ND	ND	ND	ND	ND	ND	ND	ND	ND	ND	ND
	pb	ND	ND	ND	ND	ND	ND	ND	ND	ND	ND	ND	ND	ND	ND	ND	ND
	Dfd	ND	ND	ND	ND	ND	ND	ND	ND	ND	ND	ND	ND	ND	ND	ND	ND
	Scr	ND	ND	ND	ND	ND	ND	ND	ND	ND	ND	ND	ND	ND	ND	ND	ND
	Antp	ND	ND	ND	ND	ND	ND	ND	ND	ND	ND	ND	ND	ND	ND	ND	ND
	Ubx	ND	ND	ND	ND	ND	ND	ND	ND	ND	ND	ND	ND	ND	ND	ND	ND
	abdA	ND	ND	ND	ND	ND	ND	ND	ND	ND	ND	ND	ND	ND	ND	ND	ND
	AbdB	ND	ND	ND	ND	ND	ND	ND	ND	ND	ND	ND	ND	ND	ND	ND	ND
4:50-6:00	lab	5	5	5	5	5	5	5	5	5	5	5	5	5	5	5	5
	pb	5	5	5	5	5	5	5	5	5	5	5	5	5	5	5	5
	Dfd	7	7	7	7	7	7	7	7	7	6	4	4	4	4	4	4
	Scr	7	7	7	7	7	7	7	7	7	6	4	4	4	4	4	4
	Antp	12	12	12	12	12	12	12	12	12	11	9	9	9	9	9	9
	Ubx	9	8	8	8	8	8	8	8	8	8	9	9	9	9	9	9
	abdA	9	8	8	8	8	8	8	8	8	8	9	9	9	9	9	9
	AbdB	9	8	8	8	8	8	8	8	8	8	9	9	9	9	9	9
6:00-7:20	lab	5	5	5	5	5	5	5	5	5	5	5	5	5	5	5	5
	pb	5	5	5	5	5	5	5	5	5	5	5	5	5	5	5	5
	Dfd	7	7	7	7	7	7	7	7	7	7	7	7	7	7	7	7
	Scr	7	7	7	7	7	7	7	7	7	7	7	7	7	7	7	7
	Antp	12	12	12	12	12	12	12	12	12	12	12	12	12	12	12	12
	Ubx	8	9	9	9	9	9	9	9	9	9	9	9	9	9	9	9
	abdA	8	9	9	9	9	9	9	9	9	9	9	9	9	9	9	9
	AbdB	8	9	9	9	9	9	9	9	9	9	9	9	9	9	9	9
7:20-12:00	lab	7	7	6	6	7	7	7	7	7	7	7	7	7	7	7	7
	pb	7	7	6	6	7	7	7	7	7	7	7	7	7	7	7	7
	Dfd	15	14	16	15	16	16	16	16	16	16	16	16	16	16	16	12
	Scr	15	14	16	15	16	16	16	16	16	16	16	16	16	16	16	12
	Antp	22	21	22	21	23	23	23	23	23	23	23	23	23	23	23	19
	Ubx	17	6	16	16	17	17	17	17	17	17	17	17	17	17	17	15
	abdA	17	6	16	16	17	17	17	17	17	17	17	17	17	17	17	15
	AbdB	17	6	16	16	17	17	17	17	17	17	17	17	17	17	17	15

Supplementary Table 8: Number of distances (BX-C) measured in Figure 3-5

			Head_PS0		PS2-PS4		PS9-PS12		PS14	
			No. dist	No. emb	No. dist	No. emb	No. dist	No. emb	No. dist	No. emb
3:50-4:50	Control_Ph del	Ubx-AbdB	7760	14	6622	10	3642	7	1475	6
		abdA-AbdB	7737		6578		3631		1480	
		Ubx-abdA	7795		6716		3551		1461	
	Ph del	Ubx-AbdB	6999	13	5697	9	5001	8	1705	6
		abdA-AbdB	6986		5677		5046		1738	
		Ubx-abdA	7043		5712		4881		1697	
	Control_Pc xt109	Ubx-AbdB	5215	10	4507	6	5461	7	1836	6
		abdA-AbdB	5122		4437		5279		1793	
		Ubx-abdA	5311		4592		5494		1967	
	Pc xt109	Ubx-AbdB	9036	13	9690	11	3513	5	1645	5
		abdA-AbdB	8876		9409		3537		1641	
		Ubx-abdA	9002		9505		3542		1688	
4:50-6:00	Control_Ph del	Ubx-AbdB	8644	11	7089	6	6373	8	2181	8
		abdA-AbdB	8603		7036		6408		2165	
		Ubx-abdA	8599		7043		6085		2148	
	Ph del	Ubx-AbdB	8422	14	7936	10	8762	9	3622	7
		abdA-AbdB	8479		8148		8595		3617	
		Ubx-abdA	8667		8134		8721		3683	
	Control_Pc xt109	Ubx-AbdB	5483	9	4947	6	4693	5	1505	5
		abdA-AbdB	5400		4806		4604		1523	
		Ubx-abdA	5433		4877		4833		1596	
	Pc xt109	Ubx-AbdB	7376	11	9236	8	6585	7	2335	5
		abdA-AbdB	7389		9297		6506		2331	
		Ubx-abdA	7589		9585		6621		2390	
6:00-7:20	Control_Ph del	Ubx-AbdB	6268	10	9521	8	3626	5	842	4
		abdA-AbdB	6239		9556		3519		829	
		Ubx-abdA	6311		9645		3543		849	
	Ph del	Ubx-AbdB	8489	15	10459	8	9474	9	4229	7
		abdA-AbdB	8637		10704		9748		4419	
		Ubx-abdA	8720		10927		9662		4390	
	Control_Pc xt109	Ubx-AbdB	4338	11	6941	8	6344	9	2269	6
		abdA-AbdB	4337		7212		6272		2205	
		Ubx-abdA	4553		7503		6554		2334	
	Pc xt109	Ubx-AbdB	6980	12	6366	8	13503	10	5051	10
		abdA-AbdB	6986		6336		13362		5130	
		Ubx-abdA	7064		6400		13602		5163	
7:20-12:00	Control_Ph del	Ubx-AbdB	7942	12	10980	12	9242	11	1293	9
		abdA-AbdB	7799		10942		8979		1259	
		Ubx-abdA	7825		10990		8779		1308	
	Ph del	Ubx-AbdB	4535	13	7205	12	9776	13	515	6
		abdA-AbdB	4498		7332		10088		539	
		Ubx-abdA	4703		7545		10308		576	
	Control_Pc xt109	Ubx-AbdB	6788	11	7328	11	7856	11	1243	7
		abdA-AbdB	6641		7177		7627		1204	
		Ubx-abdA	6851		7479		7823		1241	
	Pc xt109	Ubx-AbdB	8081	17	9922	17	13259	17	1993	13
		abdA-AbdB	8032		9910		13047		1983	
		Ubx-abdA	8259		10150		13462		2042	

Supplementary Table 9: Number of distances (ANT-C) measured in Figure 3-5

			Head		PS4-PS5	
			No. dist	No. emb	No. dist	No. emb
3:50-4:50	Control_Ph del	lab-Antp	4162	12	3431	9
		Scr-Antp	4172		3457	
		lab-Scr	4172		3560	
	Ph del	lab-Antp	4198	13	3289	8
		Scr-Antp	4174		3322	
		lab-Scr	4182		3523	
	Control_Pc xt109	lab-Antp	3410	10	3551	8
		Scr-Antp	3392		3520	
		lab-Scr	3344		3541	
	Pc xt109	lab-Antp	4988	13	6939	10
		Scr-Antp	5043		7089	
		lab-Scr	5165		7386	
4:50-6:00	Control_Ph del	lab-Antp	4410	11	3527	6
		Scr-Antp	4362		3436	
		lab-Scr	4298		3421	
	Ph del	lab-Antp	3957	12	5527	8
		Scr-Antp	4006		5405	
		lab-Scr	3863		5492	
	Control_Pc xt109	lab-Antp	2513	6	3094	4
		Scr-Antp	2569		3288	
		lab-Scr	2562		3326	
	Pc xt109	lab-Antp	5093	12	4918	9
		Scr-Antp	4989		4781	
		lab-Scr	4996		4891	
6:00-7:20	Control_Ph del	lab-Antp	2680	7	3126	4
		Scr-Antp	2614		3090	
		lab-Scr	2654		3176	
	Ph del	lab-Antp	4524	12	6795	8
		Scr-Antp	4522		6927	
		lab-Scr	4464		6974	
	Control_Pc xt109	lab-Antp	3460	8	4184	6
		Scr-Antp	3407		4188	
		lab-Scr	3493		4252	
	Pc xt109	lab-Antp	3577	8	4646	6
		Scr-Antp	3641		4678	
		lab-Scr	3438		4520	
7:20-12:00	Control_Ph del	lab-Antp	5609	14	6855	14
		Scr-Antp	5603		6846	
		lab-Scr	5732		7117	
	Ph del	lab-Antp	7274	15	7246	16
		Scr-Antp	7340		7214	
		lab-Scr	7527		7434	
	Control_Pc xt109	lab-Antp	4041	12	7032	13
		Scr-Antp	4069		6953	
		lab-Scr	3980		7011	
	Pc xt109	lab-Antp	4263	14	8995	15
		Scr-Antp	4149		8621	
		lab-Scr	4134		8759	

Supplementary Table 10: Number of foci analysed in Figure 5g, h

	Pc								Ph							
	Scr				abdA				Scr				abdA			
	cont Ph del		Ph del		cont Ph del		Ph del		cont Pc xt109		Pc xt109		cont Pc xt109		Pc xt109	
	Head	PS2	Head	PS2	Head-PS0	PS9-PS12	Head-PS0	PS9-PS12	Head	PS2	Head	PS2	Head-PS0	PS9-PS12	Head-PS0	PS9-PS12
No. emb	6	6	7	7	7	6	7	7	8	8	9	9	9	9	10	10
No. foci	782	348	773	256	873	600	571	602	715	533	743	504	872	724	649	624

Supplementary Table 11: Number of distances measured in Supplementary Figure 8

			Ubx-AbdB	abdA-AbdB	Ubx-abdA	lab-Antp	Scr-Antp	lab-Scr
3:50-4:50	repressed	No. dist	12975	12859	13106	7572	7564	7516
		No. emb	24			22		
	Ph del	No. dist	6999	6986	7043	4198	4174	4182
		No. emb	13			13		
	Pc xt109	No. dist	9036	8876	9002	4988	5043	5165
		No. emb	13			13		
	expressed	No. dist	8698	3273	8932	6982	6977	8327
		No. emb	16	12	16	17	17	17
7:20-12:00	repressed	No. dist	14730	14440	14676	9650	9672	9712
		No. emb	23			26		
	Ph del	No. dist	4535	4498	4703	7274	7341	7527
		No. emb	13			15		
	Pc xt109	No. dist	8081	8032	8259	4263	4149	4134
		No. emb	17			14		
	expressed	No. dist	13095	2463	12813	13887	13799	6954
		No. emb	22	23	22	27	27	26

Supplementary Table 12: Number of embryos analysed in Supplementary Figure 9-10

		Head	PS0	PS1	PS2	PS3	PS4	PS5	PS6	PS7	PS8	PS9	PS10	PS11	PS12	PS13	PS14
Control_Ph del	BX-C	25	24	20	16	16	16	14	14	14	14	14	15	15	15	15	14
	ANT-C	23	18	15	15	15	15	15	14	15	14	9	9	9	9	9	9
Ph del	BX-C	27	24	19	17	16	17	17	17	16	17	17	17	16	15	15	13
	ANT-C	25	15	16	16	16	16	15	15	15	14	10	10	10	10	10	10
Control_Pc xt109	BX-C	18	13	11	11	11	11	11	10	10	10	11	11	11	12	12	11
	ANT-C	16	12	12	12	12	12	12	12	12	12	8	8	8	8	8	8
Pc xt109	BX-C	24	22	21	19	19	18	18	18	18	17	10	9	10	11	10	10
	ANT-C	25	20	19	19	19	19	19	18	18	17	8	8	8	8	8	8
Control_Ph del	BX-C	12	8	12	11	12	12	11	11	11	11	11	11	10	10	10	9
	ANT-C	14	7	14	9	14	14	13	13	13	14	14	12	11	11	11	6
Ph del	BX-C	13	8	12	7	12	11	11	11	11	11	12	12	12	12	10	6
	ANT-C	15	8	13	11	16	16	16	16	15	15	15	15	16	16	16	13
Control_Pc xt109	BX-C	11	9	11	11	11	11	11	11	11	11	11	10	10	11	11	7
	ANT-C	12	5	8	9	13	13	13	13	13	13	13	13	13	13	11	10
Pc xt109	BX-C	17	8	14	13	17	17	16	17	16	16	16	17	17	15	15	13
	ANT-C	14	6	13	15	15	15	15	15	15	15	15	15	15	15	13	11

Supplementary Table 13: Number of foci analysed in Supplementary Figure 11

	Pc		Ph	
	cont Ph del	Ph del	cont Pc xt109	Pc xt109
No. of embryos	7	7	9	11
No. of foci	2170	1755	1755	1978
