# Supplementary material for: Loss of PRC1 induces higher-order opening of Hox loci independently of transcription during Drosophila embryogenesis
Source: Nat Commun. 2018 Sep 25;9:3898. doi: 10.1038/s41467-018-05945-4 (PMC6156336; doi:10.1038/s41467-018-05945-4)
Supplement: Supplementary file 3 — Description of Additional Supplementary Files [file 41467_2018_5945_MOESM3_ESM.pdf]

Description of Additional Supplementary Files

File Name: Supplementary Movie 1

Description: This video abstract describes the role of the canonical PRC1 Polycomb complex in maintaining silencing of Hox genes. It then introduces the question that was addressed in the present work, namely whether chromatin compaction, which is detected at inactive Hox genes reflects a direct function of PRC1 or whether it is the consequence of PRC1-dependent transcriptional inhibition. The approach used in this work is then described, along with the main observations. Finally, the video presents the main conclusion from the present work, namely that PRC1-dependent chromatin compaction precedes transcriptional effects.
